# Astrocytic Ephrin-B1 Regulates Oligodendrocyte Development and Myelination

**DOI:** 10.1080/17590914.2024.2401753

**Published:** 2024-10-22

**Authors:** Samantha N. Sutley-Koury, Alyssa Anderson, Christopher Taitano-Johnson, Moyinoluwa Ajayi, Anna O. Kulinich, Kimberly Contreras, Jasmin Regalado, Seema K. Tiwari-Woodruff, Iryna M. Ethell

**Affiliations:** a Division of Biomedical Sciences and Biomedical Sciences Graduate Program, School of Medicine, University of California Riverside, Riverside, California, USA; b Neuroscience Graduate Program, University of California Riverside, Riverside, California, USA

**Keywords:** Astrocyte, corpus callosum, development, ephrin, hippocampus, myelin, oligodendrocyte

## Abstract

Astrocytes have been implicated in oligodendrocyte development and myelination, however, the mechanisms by which astrocytes regulate oligodendrocytes remain unclear. Our findings suggest a new mechanism that regulates astrocyte-mediated oligodendrocyte development through ephrin-B1 signaling in astrocytes. Using a mouse model, we examined the role of astrocytic ephrin-B1 signaling in oligodendrocyte development by deleting ephrin-B1 specifically in astrocytes during the postnatal days (P)14-P28 period and used mRNA analysis, immunohistochemistry, and mouse behaviors to study its effects on oligodendrocytes and myelination. We found that deletion of astrocytic ephrin-B1 downregulated many genes associated with oligodendrocyte development, myelination, and lipid metabolism in the hippocampus and the corpus callosum. Additionally, we observed a reduced number of oligodendrocytes and impaired myelination in the corpus callosum of astrocyte-specific ephrin-B1 KO mice. Finally, our data show reduced motor strength in these mice exhibiting clasping phenotype and impaired performance in the rotarod test most likely due to impaired myelination. Our studies provide new evidence that astrocytic ephrin-B1 positively regulates oligodendrocyte development and myelination, potentially through astrocyte-oligodendrocyte interactions.

## Introduction

Astrocytes and oligodendrocytes are two major macro glial cell types in the CNS. Astrocytes perform a number of critical neuronal functions including synapse formation and elimination, synaptic plasticity, ion homeostasis, neurotransmission, as well as regulate the innate and adaptive immune responses (Khakh & Deneen, 2019; Liddelow & Barres, 2017). The primary function of oligodendrocytes is to generate the myelin sheath around axons of CNS neurons (Baumann & Pham-Dinh, 2001; De Robertis et al., 1958). Although astrocytes and oligodendrocytes play distinct roles in supporting neuronal functions, there are active interactions between these cell types. Astrocytes are suggested to be important regulators of oligodendrocyte development and function (Clemente et al., 2013). Astrocyte-secreted factors such as platelet derived growth factor (PDGF) and leukemia inhibitory factor-like (LIF) protein are required for proper oligodendrocyte development and survival (Gard et al., 1995; Raff et al., 1988;). Additionally, astrocyte-secreted factors regulate oligodendrocyte differentiation and myelination (Ishibashi et al., 2006; Richardson et al., 1988). Not only do astrocytes regulate oligodendrocytes through secreted factors, but they also influence oligodendrocyte metabolism through gap junction coupling, allowing for direct transfer of metabolites, as well as electrical coupling between the cells (Kamasawa et al., 2005; Kleopa et al., 2004; Stadelmann et al., 2019). Our findings suggest a new mechanism that regulates astrocyte-mediated oligodendrocyte development through ephrin-B/EphB receptor signaling.

Neuronal Ephs and ephrins regulate a number of neurodevelopmental processes including axon guidance, cell migration, and synapse maturation. The trans-synaptic functions of Ephs and ephrins are particularly well studied in excitatory synapses where Ephs and ephrins regulate dendritic spine formation, AMPAR and NMDAR trafficking and localization, and synaptic plasticity (Contractor et al., 2002; Dalva et al., 2000; Ethell et al., 2001; Henkemeyer et al., 2003; Kayser et al., 2006; Moeller et al., 2006; Sloniowski & Ethell, 2012). We previously observed that astrocytes express ephrin-B1, and that ephrin-B1 in astrocytes regulates both excitatory and inhibitory synapse development in the CA1 hippocampus (Nguyen et al., 2020) and astrocyte immunoreactivity following traumatic brain injury (Nikolakopoulou et al., 2016). Here we propose a new role for astrocytic ephrin-B1 signaling in oligodendrocyte development that has not been previously described. Oligodendrocyte precursor cells (OPCs) express several Ephs and ephrins, including EphA2, -A4, -B1, and -B2, and ephrin-A1, -A5, -B1, -B2, and -B3 (Linneberg et al., 2015). Moreover, EphA4 and EphB1 mRNA and protein levels in particular significantly increase as OPCs mature into oligodendrocytes (Linneberg et al., 2015). The same group also showed that EphB forward signaling in oligodendrocytes impaired myelination, while ephrin-B reverse signaling in oligodendrocytes enhanced myelination, supporting a role for ephrin-B/EphB in regulating oligodendrocyte differentiation and myelination (Linneberg et al., 2015). Additionally, myelinating oligodendrocytes were shown to express ephrin-B3, which acts as an inhibitor of neurite outgrowth during development, by interacting with Eph receptors in cortical neurons (Benson et al., 2005). Furthermore, both ephrin-B and ephrin-A signaling in OPCs controls OPC adhesion and migration (Chatzopoulou et al., 2004). Treatment of OPCs with ephrin-Bs showed that ephrin-B1 and ephrin-B3 inhibit OPC differentiation in culture by activating EphA4 signaling in OPCs, leading to inhibition of focal adhesion (FAK), which was previously described as a positive regulator of OPC development and myelination (Forrest et al., 2009; Syed et al., 2016). These results were further corroborated by another group showing that EphA4 signaling in oligodendrocytes prevented axo-glial contact formation and myelination (Harboe et al., 2018). Together, these findings support a role for Eph/ephrin signaling in oligodendrocyte development and myelination, however the contributions of astrocyte specific ephrin-B1 in regulating these processes has not been previously described.

The goal of this study was to determine the role of astrocytic ephrin-B1 in astrocyte-oligodendrocyte communications and oligodendrocyte development. We targeted the postnatal day (P)14-P28 period of oligodendrocyte development and myelination and examined the effects of ephrin-B1 deletion from astrocytes. We observed a significant downregulation in genes implicated in oligodendrocyte differentiation and myelination using mRNA analysis. We also found reduced levels of MBP and Olig2-positive cells, but increased GFAP immunoreactivity in the corpus callosum of astrocyte-specific ephrin-B1 KO mice. In addition, we report impaired motor strength in these mice showing clasping phenotype and impaired performance in rotarod test most likely due to impaired myelination. Our findings provide new evidence for the role of astrocytic ephrin-B1 in regulating oligodendrocyte development and myelination.

## Methods

### Ethics Statement

Mouse studies were performed according to National Institutes of Health and Institutional Animal Care and Use Committee at the University of California Riverside guidelines; animal welfare assurance #A3439-01 is on file with the Office of Laboratory Animal Welfare. Mice were maintained in an Association for Assessment and Accreditation of Laboratory Animal Care-accredited facility under 12 h light/dark cycle and fed standard mouse chow.

### Mice

In order to evaluate the effects of astrocytic ephrin-B1 deletion on myelination, and oligodendrocyte development, we used two mouse lines: (1) ERT2-Cre*^GFAP^ephrin-B1*^flox/y^ KO mice were generated by breeding female *ephrin-B1*^flox/flox^ (129S-*Efnb1* ^flox^/J, RRID:IMSR_JAX:007664) mice with male ERT2-Cre*^GFAP^* (B6.Cg-Tg(*GFAP*-cre/ERT2)505Fmv/J, RRID:IMSR_JAX: 012849) mice. *Ephrin-B1*^flox/y^ mice that did not express ERT2-CreGFAP were used as controls (2) Rosa-CAG-LSL-tdTomato reporter mice (CAG-tdTomato; RRID:IMSR_JAX:007909) were bred with ERT2-Cre*^GFAP+/+^* (B6.Cg-Tg(*GFAP*-cre/ERT2)505Fmv/J RRID:IMSR_JAX:012849) mice to generate tdTomatoERT2-Cre*^GFAP^* mice first. Then, male tdTomatoERT2-Cre*^GFAP^* mice were crossed with female *ephrin-B1*^flox/flox^ to obtain tdTomatoERT2-Cre*^GFAP^ephrin-B1*^flox/y^ KO male mice. For controls, tdTomatoERT2-Cre*^GFAP^* male mice were crossed with tdTomatoERT2-Cre*^GFAP^* female mice to generate tdTomatoERT2-Cre*^GFAP^* control male mice, resulting in tdTomato expression in astrocytes (used for nanostring/mRNA analysis). Both control and KO mice were treated with tamoxifen at P14 intraperitoneally (0.5 mg in 5 mg/ml of 1:9 ethanol/sunflower seed oil solution) once a day for 5 consecutive days and analysis was performed at P28.

### Immunohistochemistry

Immunohistochemistry was performed as previously described in Yamate-Morgan et al. (2019) and Nguyen et al. (2020). Animals were anesthetized with isoflurane and transcardially perfused with 0.1 M PBS followed by fixation with 4% paraformaldehyde (PFA) in 0.1 M PBS, pH 7.4. Brains were postfixed for 2 h in 4% PFA in 0.1 M PBS. 100 μm coronal brain slices were obtained via vibratome sectioning. Samples were washed in PBS, permeabilized in 0.3% Triton in PBS for 30 min, blocked for 2 h in 15% normal goat serum (NGS) in 0.1 M PBS. Primary and secondary antibodies were diluted in PBS. Samples were incubated in primary antibodies for 2 h at room temperature and then overnight at 4 °C. Chicken anti-myelin basic protein (MBP) (1:500, EMD Millipore, AB9348, RRID:AB_2140366) was used to detect myelin and rabbit anti-Olig2 (1:500, EMD Millipore, AB9610, RRID:AB_570666) was used to identify oligodendrocytes. Ephrin-B1 was detected using goat anti-ephrin-B1 antibody (1:50, R&D Systems, AF473, RRID:AB_2293419). Astrocytes were identified using rabbit anti-GFAP antibody (1:500, Cell Signaling Technology, 12389, RRID:AB_2631098). Following incubation with primary antibody, samples were washed with PBS, then incubated with secondary antibodies for 2 h at room temperature. Primary antibodies were recognized with the following secondary antibodies: donkey anti-rabbit 488 IgG (1:500, Invitrogen, A-21206, RRID:AB_2535792) and goat anti-chicken 647 (1:500, Invitrogen, A-21449, RRID:AB_2535866), or donkey anti-rabbit IgG 594 (1:500, Invitrogen, A-21207, RRID:AB_141637) and donkey anti-goat IgG 488 (1:500, Invitrogen, A-11055, RRID:AB_2534102),. Samples were then washed in PBS and then mounted on coverslips with Vectashield antifade mounting medium with Dapi (Vector Laboratories, H-2000).

### Confocal Imaging and Analysis

Confocal imaging and analysis was performed as previously with modifications (Nguyen et al., 2020). Confocal images of coronal brain slices containing the stratum oriens (SO), stratum pyramidal (SP), and stratum radiatum (SR) of the dorsal CA1 hippocampus or corpus callosum (CC) were taken using a Zeiss LSM 880 inverted laser scanning microscope. High resolution optical sections were acquired with a 20x air objective (0.8 NA), 1x zoom at 1 μm step intervals, (1024x1024) pixel format to measure myelin basic protein (MBP) intensity, the number of GFAP-positive processes and GFAP immunoreactivity, the number of Olig2-positive oligodendrocytes, and Olig2 immunoreactivity. Identical conditions were used to acquire and process all samples for analysis.

For analysis of MBP intensity, Z-stacks of equal size were collapsed into a single image by projection and converted to a tiff file, images were separated by channel and regions of interest (ROIs) were drawn around the area to be measured. For CC measurement the ROI was drawn around the CC and for the hippocampus, ROIs contained SO, SP, and SR layers. Any cortical areas in the image were excluded from the measurements. Mean fluorescence intensity levels within the ROIs were measured. Background signal was measured using a secondary antibody only control and then was subtracted from the fluorescence intensity measurement. Statistical analysis was performed using GraphPad Prism 10 software (RRID:SCR_002798) using a two-tailed student’s t-test.

For analysis of Olig2+ cell density and GFAP + processes, Z-stacks of equal size were collapsed into a single image by projection, converted to a tiff file, separated by channel, and background subtracted. Each image was threshold-adjusted, converted into a binary image, the despeckle function was used to eliminate noise and the watershed function was used to segment overlapping cells. Particle analysis was used to count the number of cells or processes per image and to generate ROIs around the counted cells. Images were re-opened, separated by channel, and the ROIs were applied to the Olig2 channel. Mean fluorescence intensity levels within the ROIs were measured. Background signal was measured using a secondary antibody only control and then was subtracted from the fluorescence intensity measurement. Statistical analysis was performed using GraphPad Prism 10 software (RRID:SCR_002798) using a two-tailed student’s t-test.

Analysis of ephrin-B1 levels in tdTomato-expressing astrocytes was performed in ImageJ software. For the analysis, single image planes were separated by channel, ROIs were drawn around astrocytes’ soma and then ephrin-B1 mean fluorescence intensity levels within the ROIs were measured. The background signal was measured using a secondary antibody only control and then was subtracted from the fluorescence intensity measurement.

### Nanostring

Half-brains were dissected and frozen in liquid nitrogen and stored in -80 °C. RNAlater Ice (Invitrogen, AM7030) was used to thaw samples according to manufacturer’s instructions. After thawing in RNAlater Ice, hippocampal tissue was dissected in DEPC-treated PBS on ice. Following dissection, total RNA was isolated using Invitrogen PureLink RNA Mini Kit (ThermoFisher Scientific/Invitrogen, 12183020). Samples were lysed in RNA lysis buffer with β-mercaptoethanol (BME) added as described in the protocol provided by the kit. Tissue samples were homogenized in a lysis buffer provided by the kit using a pestle and then were passed through an 18-gauge needle. Following lysis, samples were centrifuged at 12,000 *g* for 2 min and the supernatants were transferred to fresh tubes. RNA isolation was then performed according to the manufacturer’s instructions. Samples were eluted in nuclease free water. To remove any residual salts or contaminants from buffers used during RNA isolation, RNA samples were further purified by ethanol precipitation as follows; 0.1 volumes of 0.3 M Na-Acetate and 3 volumes of ice-cold 100% ethanol were added to the samples. Samples were incubated at -20 °C overnight, centrifuged at 12,000 *g*, and then ethanol was removed, the pellet was dried, and samples were reconstituted in nuclease free water. RNA concentration and purity was determined using a Nanodrop spectrophotometer (RRID:SCR_016517), then samples were analyzed using Agilent 2100 Bioanalyzer/Advanced Analytics Fragment Analyzer (RRID:SCR_018043). RIN values above 7 were considered to be adequate for Nanostring profiling. RNA samples were analyzed using nCounter Mouse Glial Profiling Panel (Nanostring, XT-CSO-M-GLIAL-12) according to manufacturer’s instructions. Briefly, 50 ng of unamplified RNA was hybridized with the reporter codeset at 65 °C for 18 h. Samples were spun down, nuclease free water was added to the samples, and then the samples were loaded into the nCounter cartridge. The cartridge was run on the Nanostring nCounter SPRINT Profiler (RRID:SCR_021712). Data was exported and analyzed using Nanostring nSolver and Advanced Analysis Software (RRID:SCR_021712). Normalized linear counts for all genes in the panel were used to graph fold changes of control and KO genes. Statistical analysis was performed in the Nanostring online advanced analysis differential expression module using the differential expression algorithm described in the advanced analysis manual (nCounter Advanced Analysis 2.0 User Manual, MAN-10030-03). Differentially expressed genes were assigned to functional categories by Nanostring software and were presented based on the category assigned by Nanostring. The Bonferroni correction was used to correct for the high number of multiple comparisons.

### qRT-PCR Analysis

P28 mouse brains were dissected on ice in DEPC-treated PBS. Following dissection, total RNA was acutely isolated as described above. Samples were eluted in nuclease free water, and RNA concentration and purity was determined using a Nanodrop spectrophotometer (RRID:SCR_016517). Samples were diluted to equal concentrations in nuclease free water. cDNA was synthesized using a High-Capacity cDNA Reverse Transcription Kit (Applied Biosystems, 4368814). qRT-PCR was performed using PowerSYBR Green PCR Master Mix (Applied Biosystems, 4367659) on Quantstudio 6 Flex System thermocycler (Applied Biosystem/Life Technologies, RRID:SCR_020239). Data was normalized to GAPDH loading control and then analyzed using _ΔΔ_Ct method normalized to GAPDH housekeeping gene. Statistical analysis performed in GraphPad Prism 10 software using two-tailed student’s t-test. Primer sequences are listed in Supplemental Materials (Table S1).

### Western Blot Analysis

The corpus callosum was dissected from individual animals (n = 10: 5 wild-type and 5 knockout), flash frozen with dry ice, and stored at -80 °C. Protein lysates were prepared by homogenization of samples in a lysis buffer cocktail containing RIPA buffer supplemented with a protease inhibitor (1:500 dilution), and phosphatase inhibitor (1:500 dilution). Following homogenization, supernatants were collected, aliquoted, and stored at -80 °C. Concentration of protein content was determined with the Pierce^™^ Microplate BCA Protein Assay Kit (ThermoFisher Scientific, Rockford, IL). Prior to electrophoresis, 20 µg of protein from each sample was combined with 4X Laemmli Buffer with SDS (ThermoFisher Scientific, Ward Hill, MA) and water, then denatured at 70 °C for 10 minutes. A standard (Precision Plus Protein Dual Color Standards; BIORAD, USA) and samples were run on 4–20% precast polyacrylamide gel (BIO-RAD, USA) then transferred to a PVDF membrane (BIO-RAD, USA) overnight at 4 °C at 30 V. After transfer completion, membranes were blocked with 1X Tris buffered saline (1X TBS; 50 mM Tris–HCl pH 7.4 and 150 mM NaCl) containing 5% powdered milk. Blots were incubated with the following primary antibodies overnight at 4 °C in blocking buffer containing 1X TBS with 0.1% Tween 20 (TBST) and 5% powdered milk: polyclonal rabbit anti-Myelin Basic Protein (Abcam; 1:2,000; ∼18.5 kDa & 21.5 kDa); monoclonal mouse anti-Glyceraldehyde-3-Phosphate Dehydrogenase (EMD Millipore; 1:50,000; 34 kDa). Blots were washed three times in TBST then incubated in horseradish peroxidase-linked secondary antibodies (Cell Signaling, anti-mouse/rabbit IgG HRP-linked antibody; 1:1,000) against the primary antibody host and a StrepTactin horseradish peroxidase-conjugated secondary antibody (BIORAD, Precision Protein StrepTactin-HRP Conjugate; 1:10,000) against the standard for 1 h at room temperature followed by three TBST washes. Membranes were incubated with an enhancer reagent (Clarity Western ECL Substrate; BIORAD, USA) for 1 minute, then imaged using a ChemiDoc (BIORAD, USA). Quantification of expression levels was done by comparison of protein of interest band density to glyceraldehyde phosphate dehydrogenase (GAPDH) band density. Quantification graphs are from 3 wildtype (WT) and 5 KO animals. GraphPad Prism 10 software using a two-tailed student’s t-test.

### Clasping

Before testing, mice were housed in a room with a 12 h light/dark cycle and ad libitum access to food and water. Cages were transferred to the behavioral room 30 min prior to testing for habituation. Clasping behavior was assessed with methods similar to previously described (Guyenet et al., 2010). Briefly, mice were held by the base of the tail and were video recorded as they were suspended in the air for one minute. The presence of a clasping phenotype was scored by an experimenter blind to the condition as follows: 0 – hindlimbs were consistently splayed away from the abdomen, 1 – one hindlimb was retracted toward the abdomen, 2 – both hindlimbs were retracted toward the abdomen, and 3 – both hindlimbs were retracted toward the abdomen and touched the abdomen. Statistical analysis performed in GraphPad Prism 10 software using Mann-Whitney test and Log-rank (Mantel-Cox) test.

### Rotarod Motor Performance

Cages were moved to the behavioral room 1 h prior to testing. Motor performance was assessed using the Rotarod apparatus (Med Associates, Inc., St. Albans, VT) as previously performed with modifications (Kumar et al., 2013;Tiwari-Woodruff et al., 2007). Briefly, mice were placed on the rotating horizontal rod set to a speed of 3–30 rpm for a maximum of 300 s. The amount of time the mouse was able to walk on the accelerating rod was recorded. Each mouse was tested in three trials separated by 15 min inter-trial intervals. The average of the three trials was reported as a single value for each mouse. Statistical analysis was performed in GraphPad Prism 10 software using the Mann-Whitney test.

## Results

### Deletion of Astrocytic Ephrin-B1 Affected Expression of Genes Associated with Astrocyte Metabolism, Calcium Signaling and Reactive State

**Figure 1.**
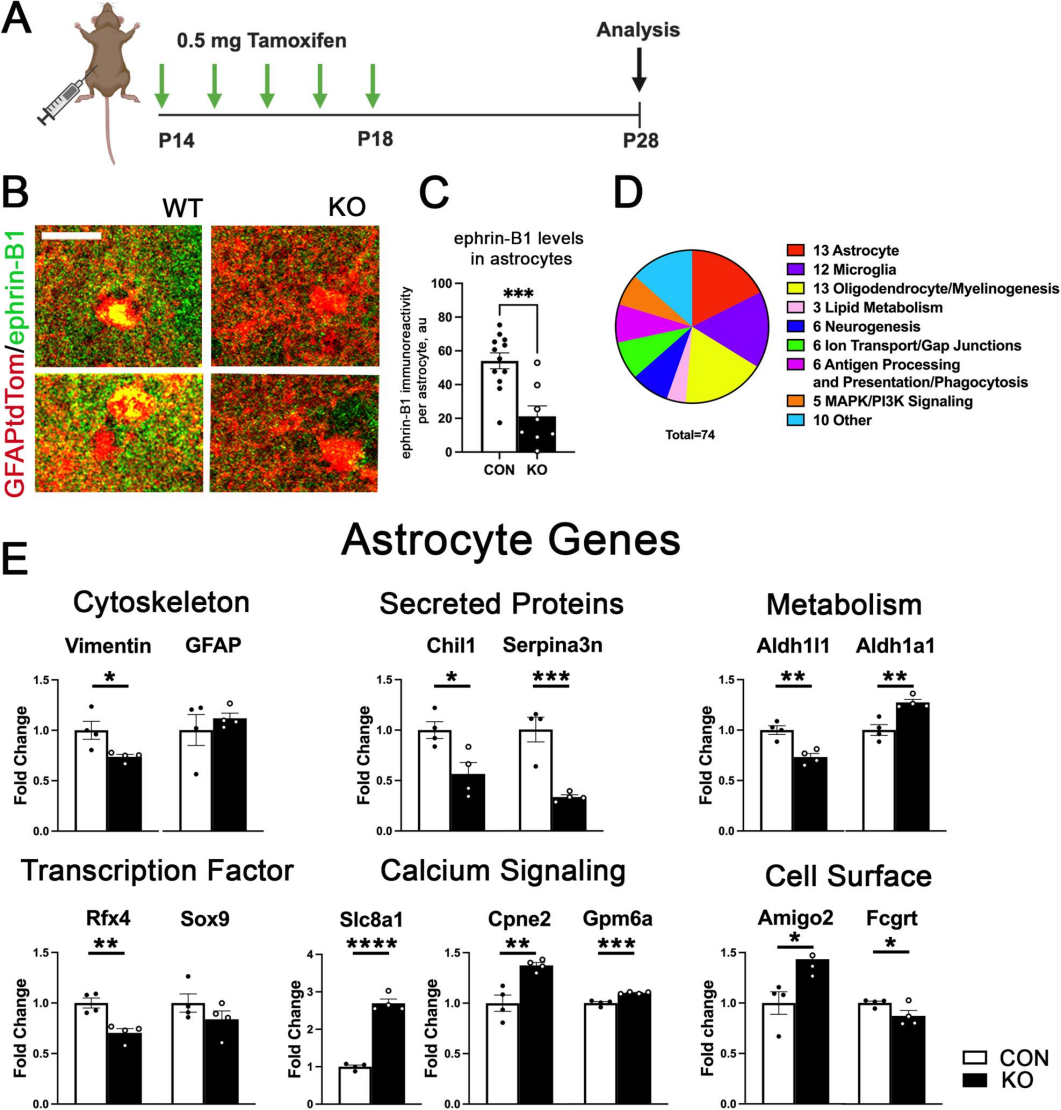
Deletion of astrocytic ephrin-B1 alters astrocyte-associated gene expression. (A) Graphic depicts the astrocyte specific ephrin-B1 knockout (KO) model and experimental timeline. (B) Confocal images show ephrin-B1 immunoreactivity (green) in td-Tomato-expressing (red) control and KO astrocytes, scale bar 25 μm. (C) Graph shows levels of ephrin-B1 immunofluorescence in control and KO astrocytes. Deletion of astrocytic ephrin-B1 significantly reduced ephrin-B1 immunoreactivity in KO astrocytes (n = 12 images from 3 mice per group, t-test, ***p < 0.001). (D) Graph shows the number of differentially expressed genes by category. Glial genes including astrocytes, oligodendrocytes, and microglia as well as myelin and lipid metabolism associated genes showed many significant differences following deletion of astrocytic ephrin-B1 KO. See complete gene analysis in Supplementary Materials (Extended Data Table 1). (E) Graphs show mRNA levels astrocyte-associated genes which were differentially expressed following astrocytic ephrin-B1 KO (n = 4 mice/group t-test, *p < 0.05, **p < 0.01, ***p < 0.001).

To investigate the effects of astrocytic ephrin-B1 on hippocampal gene expression, we conditionally deleted ephrin-B1 in astrocytes during P14-P28 developmental period (Figure 1A). Deletion of astrocytic ephrin-B1 reduced ephrin-B1 immunofluorescence intensity in astrocytes (Figure 1B-C, t-test, p = 0.00045). In order to assess whether developmental deletion of astrocytic ephrin-B1 alters the expression of glial genes, we performed Nanostring analysis on RNA isolated from brain tissues including hippocampus and CC of P28 control and KO male mice using the Nanostring Glial Profiling Panel. Gene categories were assigned in the Nanostring software and presented based on the category assigned by Nanostring. Of the 74 differentially expressed genes, 13 were associated with astrocytes (Figure 1D). Vimentin, an intermediate filament expressed in astrocytes (Schnitzer et al., 1981), was significantly reduced (Figure 1E, p = 0.0106), while GFAP was unchanged (p = 0.506). Genes encoding for *Chil1* (p = 0.0457) and *Serpina3n* (Figure 1E, p = 0.000191), both of which are secreted by reactive astrocytes (Escartin et al., 2021), were also found to be reduced in KO animals, suggesting that deletion of astrocytic ephrin-B1 may reduce the inflammatory response and or reactivity of astrocytes. *Serpina3n* is a serine protease inhibitor which is secreted into the extracellular matrix (Escartin et al., 2021). Enzymes associated with metabolism in astrocytes were also affected by KO, such as *Aldh1l1* (Figure 1E, p = 0.0033), which was reduced in KO mice and *Aldh1a1* (Figure 1E, p = 0.00615), which was increased in KO mice (Anthony & Heintz, 2007; Kwak et al., 2020). The transcription factor *Rfx4*, which was shown to be enriched in astrocytes was also reduced in KO mice (Figure 1E, 0.00242). Two genes associated with calcium signaling in astrocytes, *Cpne2* (Figure 1E, p = 0.00879) and *Gmp6* (Figure 1E, p = 0.000642) were increased in KO animals (Creutz et al., 1998; Mukobata et al., 2002). The most significantly regulated gene we detected via Nanostring analysis was the *Slc8a1* gene (Figure 1E, p = 3.47E-06), encoding the NCX sodium-calcium exchanger (Rose et al., 2020). Although this gene is not specific to astrocytes and is expressed in neurons, it is also expressed in astrocytes and plays an important role in sodium/calcium buffering in astrocytes (Rose et al., 2020). Astrocyte calcium signaling has been implicated in astrocyte functions such as release of gliotransmitters, which could have a profound impact on neuronal function (Bazargani & Attwell, 2016; Goenaga et al., 2023). Interestingly we found that expression of the cell surface receptor *Amigo-2*, which is associated with A1-polarized reactive astrocytes (Kim et al., 2024) was significantly increased (Figure 1E, p = 0.0209), while the cell surface receptor *Fcgrt* (Stamou et al., 2018) was modestly reduced (Figure 1E, p = 0.0464). A full table of the statistical analysis and levels of all genes assayed via Nanostring analysis can be found in the Supplementary Materials (Extended Data Table 1).

### Developmental Deletion of ephrin-B1 in Astrocytes Impairs Expression of Oligodendrocyte- and Myelin-Associated Genes

**Figure 2.**
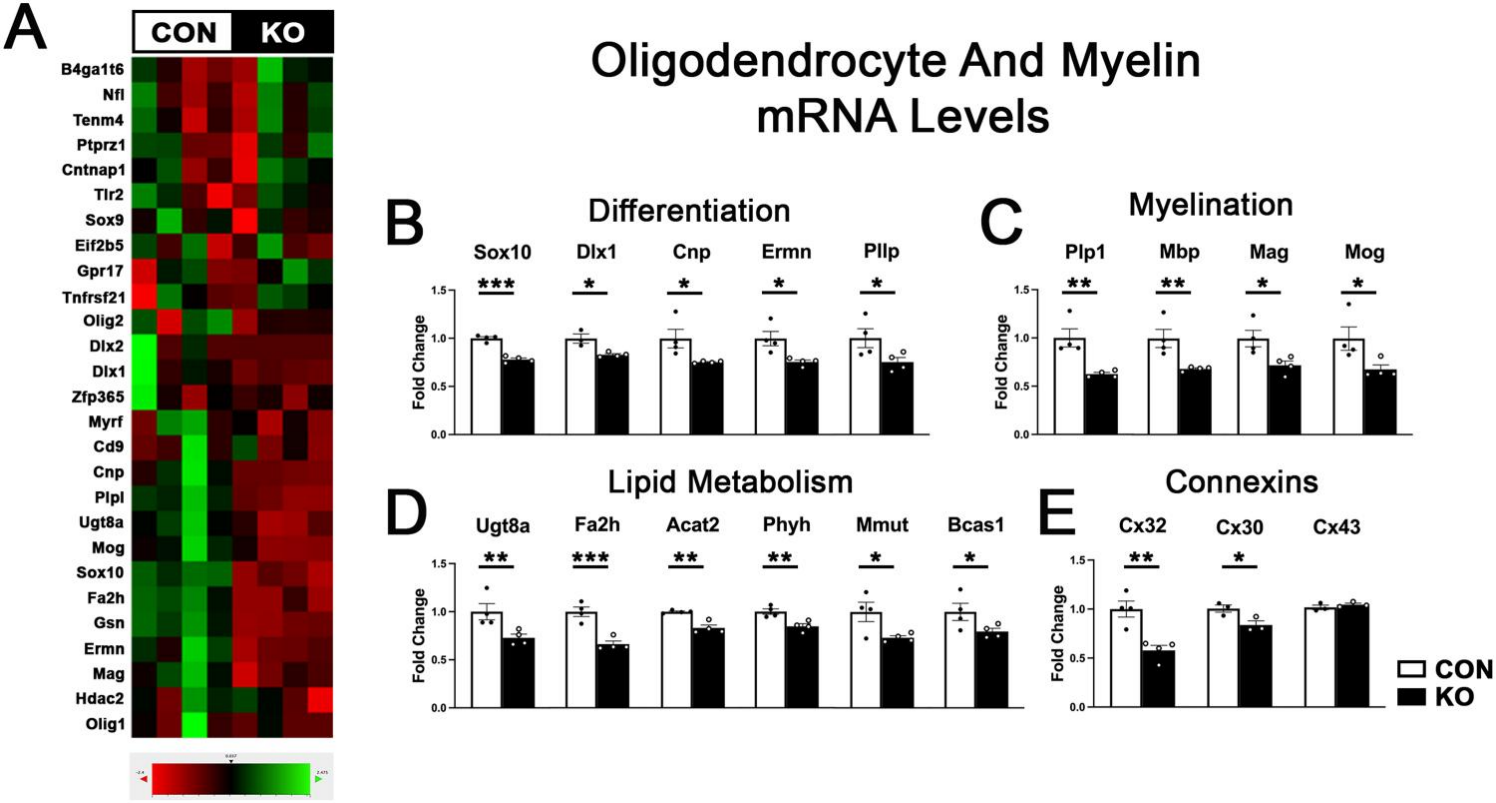
Astrocytic ephrin-B1 KO impairs gene expression of oligodendrocyte- and myelin-associated genes. (A) Heatmap of gene expression of oligodendrocyte-associated genes in control (CON) and KO animals, generated from Nanostring gene expression analysis. (B) Graphs show mRNA levels of genes associated with oligodendrocyte differentiation in CON and KO animals (n = 3-4 mice/group, *p < 0.05, ****p < 0.0001). KO mice showed significant reductions in many oligodendrocyte-related genes. (C) Graphs show mRNA levels of myelin associated genes in CON and KO animals (n = 4 mice/group, *p < 0.05, **p < 0.01). Myelin-associated gene expression was significantly reduced in KO animals. (D) Graphs show mRNA levels of genes associated with lipid metabolism in CON and KO animals (n = 4 mice/group t-test, *p < 0.05, **p < 0.01). Genes associated with lipid metabolism were also reduced following astrocytic ephrin-B1 KO. **E)** Graph shows mRNA expression of connexin genes, in CON and KO animals (n = 3-4 mice/group t-test, **p < 0.01). All data are represented as mean ± SEM.

Nanostring analysis revealed that deletion of astrocytic ephrin-B1 during the P14-P28 developmental period also affected expression of many oligodendrocyte- and myelin-associated genes. We observed a broad downregulation of genes associated with oligodendrocyte differentiation, myelination, and lipid metabolism (Figure 2B-D). mRNA levels of several transcription factors implicated in oligodendrocyte differentiation were reduced in KO mice, including *Sox10* and *Dlx1* (Figure 2B, *Sox10*: p = 0.000113; *Dlx1*: p = 0.0352), suggesting a possible reduction in oligodendrocytes or impaired oligodendrocyte maturation in KO mice (Kuhn et al., 2019; Petryniak et al., 2007; Pozniak et al., 2010; Stolt et al., 2002). We found that genes encoding the major protein components of the myelin sheath including myelin basic protein (*MBP*) and proteolipid protein 1 (*Plp1*) (Kuhn et al., 2019) were also significantly reduced in KO mice (Figure 2C, *MBP*: p = 0.00327; *Plp1*: p = 0.00115). Furthermore, we detected a reduced mRNA expression of genes involved in lipid metabolism in KO mice (Figure 2D). Of particular interest, we found reduced *Ugt8a* and *Fa2h* mRNA levels (Figure 2D, *Ugt8a*: p = 0.00877; *Fa2h*: p = 0.000862), encoding the enzymes involved in sphingolipid synthesis, which are abundant in the myelin sheath (Coetzee et al., 1998; Eckhardt, 2023; Marcus et al., 2000; Maldonado et al., 2008). The reduction in genes encoding the components of the myelin sheath and enzymes regulating lipid metabolism may suggest that myelination is likely impaired following deletion of astrocytic ephrin-B1. Interestingly, we found that expression of connexin-32 (Cx32), a connexin found in oligodendrocytes and involved in coupling oligodendrocytes and astrocytes (Altevogt & Paul, 2004; Kamasawa et al., 2005; Kleopa et al., 2004; Stadelmann et al., 2019), was also downregulated in KO mice (Figure 2E, p = 0.00151). We further investigated the levels of two connexins expressed in astrocytes, Cx30, which forms gap junctions with oligodendrocyte Cx32 (Altevogt & Paul, 2004; Kamasawa et al., 2005; Kleopa et al., 2004), and Cx43 using qRT-PCR and found that KO mice showed reduced levels of Cx30 (Figure 2E; t-test, t(4)=2.980, p = 0.0407), but not Cx43 (Figure 2E; t-test, t(4)=1.005, p = 0.3719). Reduced mRNA levels of Cx32 and Cx30 in KO mice could indicate impaired astrocyte-oligodendrocyte communication.

### Loss of Astrocytic ephrin-B1 Impaired Myelination and Reduced the Number of Oligodendrocytes in the CC but Not the Hippocampus

**Figure 3.**
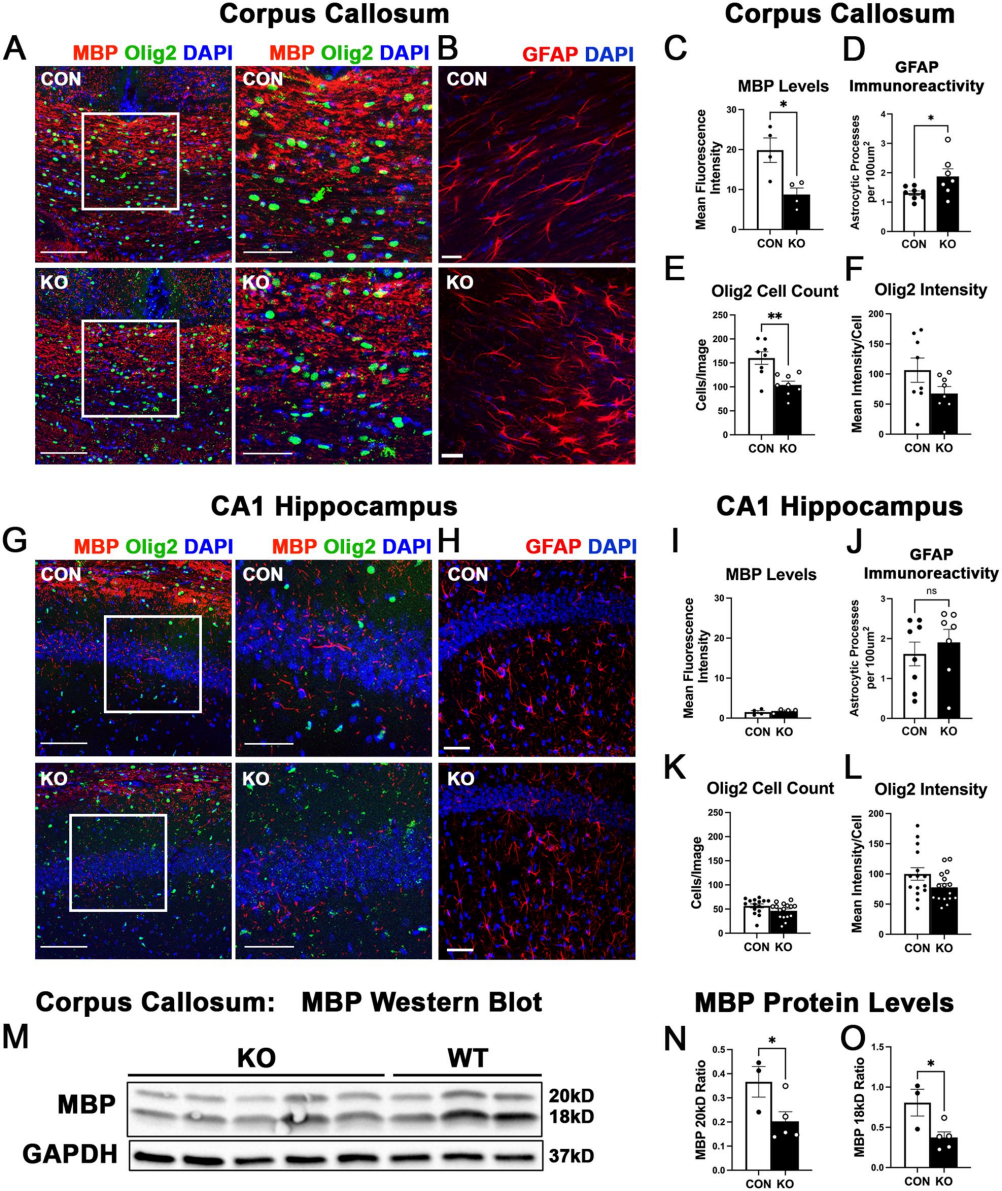
Loss of astrocytic ephrin-B1 impaired myelination and reduced the number of Olig2+ oligodendrocytes in the corpus callosum but not the hippocampus. (A) Confocal images of brain slices showing MBP (red), Olig2 (green), and Dapi (blue) immunolabeling in the corpus callosum of CON and KO mice. Scale bar, 100 μm (left) or 50 μm (right). (B) Confocal images show GFAP (red) and Dapi (blue) immunolabeling in the corpus callosum of CON and KO mice, scale bar 20 μm. (C) Graph shows the mean fluorescence intensity of MBP in the corpus callosum. KO mice showed reduced intensity of MBP compared to controls (n = 4 mice per group, 8 images per group, t-test, *p < 0.05). (D) Graph shows GFAP immunoreactivity in CON and KO mice. Deletion of astrocytic ephrin-B1 increased GFAP immunoreactivity in the corpus callosum (n = 7-8 images from 3 mice per group, t-test, *p < 0.05). (E) Graph shows the average number of Olig2+ oligodendrocytes per image of the corpus callosum of CON and KO mice. The number of Olig2+ oligodendrocytes was reduced in KO mice compared to CON (n = 4 mice per group, 8 images per group, t-test, **p < 0.001). (F) Graph shows the immunofluorescence intensity of Olig2 immunolabeling in Olig2 positive cells in the corpus callosum of CON and KO mice. Deletion of astrocytic ephrin-B1 did not affect the intensity of Olig2 immunolabeling (n = 4 mice per group, 8 images per group, t-test, p > 0.05). (G) Confocal images of brain slices depicting MBP (red), Olig2 (green), and Dapi (blue) immunolabeling in the CA1 hippocampus of CON and KO mice. Scale bar, 100 μm (left) or 50 μm (right). (H) Confocal images show GFAP (red) and Dapi (blue) immunolabeling in the CA1 hippocampus of CON and KO mice, scale bar 50 μm. (I) Graph shows the mean fluorescence intensity of MBP in the CA1 hippocampus. MBP intensity levels were unchanged in KO mice compared to controls (n = 4 mice per group, 16 images per group, t-test). (J) Graph shows GFAP immunoreactivity in CON and KO mice. Deletion of astrocytic ephrin-B1 did not affect GFAP immunoreactivity in the CA1 hippocampus (n = 7-8 images from 3 mice per group, t-test, p > 0.05). (K) Graph shows the average number of Olig2+ oligodendrocytes per image of the CA1 hippocampus of CON and KO mice. There was a non-significant trend towards a reduced density of Olig2+ oligodendrocytes in KO hippocampi compared to control hippocampi (n = 4 mice per group, 15-16 images per group, t-test, p = 0.0883). (L) Graph shows the immunofluorescence intensity of Olig2 immunolabeling in Olig2 positive cells in the CA1 hippocampus of CON and KO mice. Deletion of astrocytic ephrin-B1 did not affect the intensity of Olig2 immunolabeling (n = 4 mice per group, 15-16 images per group, t-test, p > 0.05). (M) Image shows western blot of MBP and GAPDH in astrocytic ephrin-B1 KO and CON mice. (N,O) Graphs show the 20kD (N) and the 18kD (O) MBP protein levels normalized to GAPDH. Deletion of astrocytic ephrin-B1 reduced protein levels of MBP (N,O; n = 3-5 mice, t-test, *p < 0.05). All data are represented as mean ± SEM.

Because we found such marked reductions in nearly all oligodendrocyte- and myelin-related genes assessed via nanostring, we next assessed whether myelination or oligodendrocyte development was impaired by developmental deletion of astrocytic ephrin-B1. To analyze myelin levels, we performed immunolabeling against MBP, one of the main proteins of the myelin sheath (Stadelmann et al., 2019), and analyzed the fluorescence intensity in both the CC and the CA1 hippocampus of CON and KO mice (Figure 3A, G). We found reduced MBP immunoreactivity in the CC (Figure 3C, t-test, t(6)=3.197, p = 0.0187) but not the CA1 hippocampus of KO mice (Figure 3I; t-test, t(6)=0.6161, p = 0.5605). We also analyzed GFAP immunoreactivity in the corpus callosum and CA1 hippocampus and found that GFAP immunoreactivity was increased in the corpus callosum (Figure 3D, t-test, t(13)=2.252, p = 0.0422) but unchanged in the CA1 hippocampus (Figure 3J, t-test, t(13)=0.6490, p = 0.5277). The reduced MBP levels in the CC indicates that KO mice show impaired myelination. The overall MBP immunolabeling was much lower in the hippocampus than the CC, most likely contributing to our inability to detect any differences between CON and KO mice. We also measured MBP levels using Western blot analysis and again found that deletion of astrocytic ephrin-B1 reduced MBP protein levels (Figure 3M-O; N; t(6)=2.335, p = 0.0291, O; t(6)=2.837, p = 0.0148). Next, we analyzed whether there were any differences in the oligodendrocyte development. We visualized Olig2 positive (+) oligodendrocytes using immunofluorescence labeling and counted the number of Olig2+ cells in the CC and CA1 hippocampus of CON and KO mice. Developmental deletion of astrocytic ephrin-B1 significantly reduced the number of Olig2+ cells in the CC (Figure 3E; t-test, t(14)=3.656, p = 0.0026), and resulted in a trend towards reduced numbers in the hippocampus (Figure 3K; t-test, t(29)=1.764, p = 0.0883). Although the number of oligodendrocytes was reduced in the corpus callosum of KO mice, there were no changes in the Olig2 immunoreactivity per cell in the corpus callosum (Figure 3F; t(14)=1.674, p = 0.1164) and the CA1 hippocampus (Figure 3L; t(29)=1.855, p = 0.0737), indicating that the reduced number of oligodendrocytes was unlikely to be due to our inability to detect them. Our results suggest that oligodendrocyte maturation or survival is impaired in KO mice, leading to the deficits in myelination.

### Astrocytic ephrin-B1 KO Mice Show a Clasping Phenotype and Impaired Performance in the Rotarod Test

**Figure 4.**
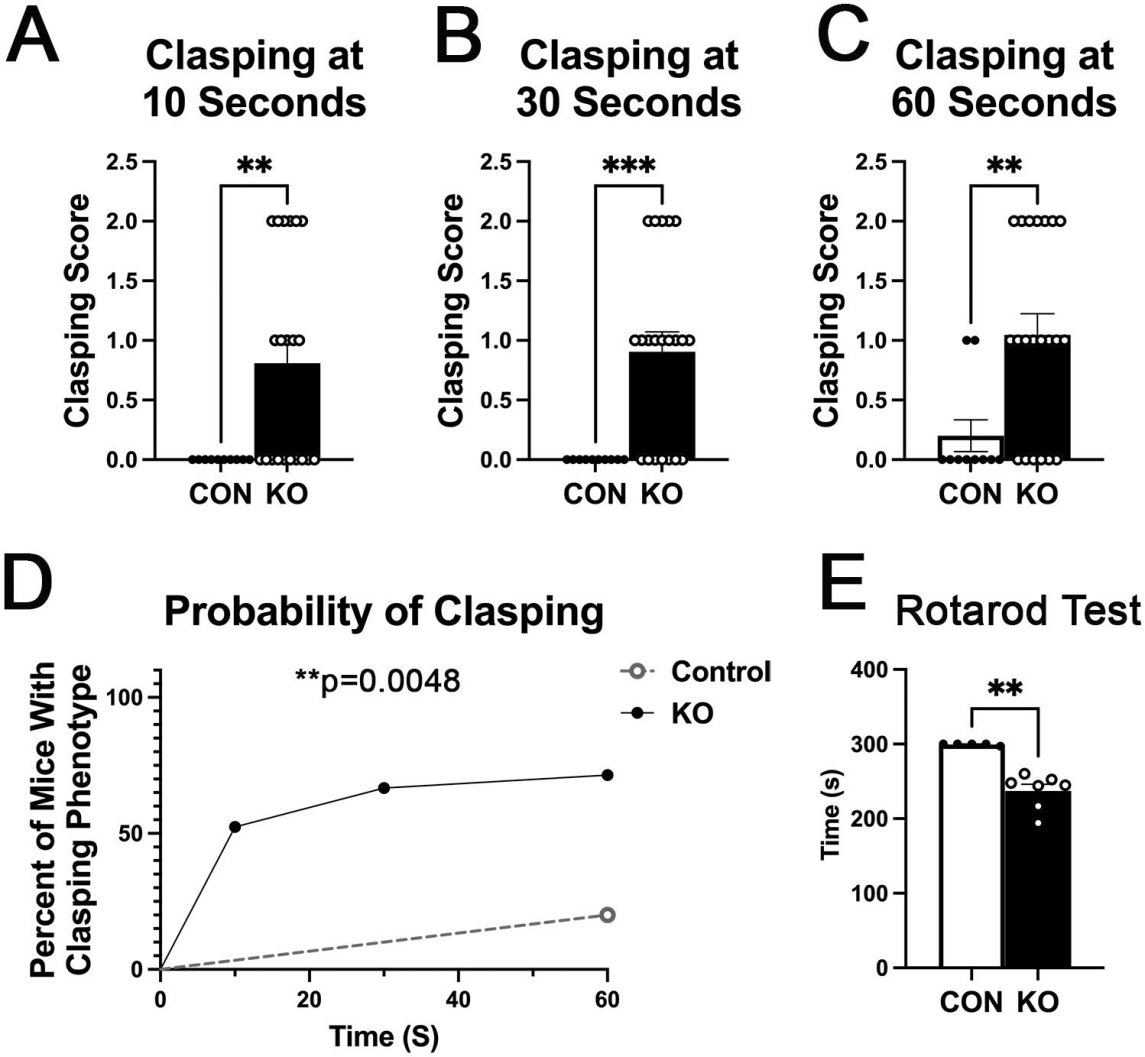
Astrocytic ephrin-B1 KO mice exhibit a clasping phenotype and impaired performance in the rotarod test. (A) Graph shows the mean clasping score at time (t)=10 s during the clasping test (n = 10-21 mice per group, Mann-Whitney test, **p < 0.001). (B) Graph shows the mean clasping score at t = 30 s during the clasping test (n = 10-21 mice per group, Mann-Whitney test, ***p < 0.0001). (C) Graph shows the mean clasping score at time (t)=60 s during the clasping test (n = 10-21 mice per group, Mann-Whitney test, **p < 0.001). KO mice exhibit a significant increase in the clasping phenotype at all three time points assessed compared to CON. (D) Graph shows the probability of developing a clasping phenotype over the course of the test. KO mice exhibited a significantly increased chance of developing a clasping phenotype over the course of the test compared to CON mice (n = 10-21 mice per group, Log-rank test, **p < 0.001). (E) Graph shows Rotarod analysis of CON and astrocytic ephrin-B1 KO mice. Astrocytic ephrin-B1 KO deletion impaired rotarod performance (n = 5-7 mice per group, Mann-Whitney test, **p < 0.001).

In order to determine whether the observed reduction in oligodendrocytes and myelin expression resulted in functional impairments in vivo, we subjected P28 CON and KO mice to a clasping test. A clasping phenotype where mice retract their hindlimbs toward the abdomen when suspended by the tail, has been observed in mouse models of EAE and the presence of a clasping phenotype can be a sign of decreased motor strength due to hypomyelination or demyelination (Bhaskaran et al., 2023; Cahill et al., 2019). We found that developmental deletion of astrocytic ephrin-B1 resulted in a clasping phenotype that was significantly increased compared to controls at all time points assessed (Figure 4A-C, t = 10 s: Mann-Whitney test, DoF = 29, p = 0.0066; t = 30 s: Mann-Whitney test, DoF = 29, p = 0.0008; t = 60 s: Mann-Whitney test, DoF = 29, p = 0.0065). Indeed, KO mice showed an increased probability of developing the clasping phenotype over the course of the test compared to controls (Figure 4D; Log-rank test, p = 0.0048). Performance in the rotarod test was also impaired by astrocytic ephrin-B1 KO (Figure 4E; Mann-Whitney test, DoF = 11, p = 0.0013). The presence of the clasping phenotype and impaired rotarod performance in KO mice is suggestive of reduced motor strength, most likely as a result of the observed deficits in oligodendrocyte development and myelination.

## Discussion

Here we provide new evidence that astrocytes regulate oligodendrocyte development and myelination during development through ephrin-B1 signaling. Our study reports that the astrocyte-specific deletion of ephrin-B1 during P14-P28 developmental period leads to (1) dysregulation of both astrocyte- and oligodendrocyte-related genes; (2) impaired oligodendrocyte development and myelination; (3) clasping phenotype and impaired rotarod performance, indicative of reduced motor strength.

We found that the developmental deletion of astrocytic ephrin-B1 led to dysregulated expression of several genes associated with reactive astrocytosis (Figure 1D-E). Of particular interest, genes encoding proteins secreted by reactive astrocytes, *Chil1* and *Serpina3n*, were found to be significantly reduced in KO animals (Figure 1E). *Chil1* (*Chi3l1*, YKL-40) plays a role in cell growth, proliferation, and survival, inflammatory response, as well as regulating synthesis and degradation of the extracellular matrix through inhibiting type 1 collagen and hyaluronic acid degradation and regulating the activity of MMPs (Zhao et al., 2020). Increased levels of *Chi3l1* in the cerebrospinal fluid (CSF) have been observed in Alzheimer’s disease (AD) and multiple sclerosis (MS) (Craig-Schapiro et al., 2010; Floro et al., 2022). Recently, *Chi3l1* overexpression was shown to reduce hippocampal neurogenesis and impair hippocampal dependent learning in mice (Jiang et al., 2023), while knockdown in astrocytes increased phagocytosis of pHrodo beads and Aβ42 peptides in culture (Lananna et al., 2020). *Serpina3n* has been shown to be increased in many neuroinflammatory contexts, including AD, stroke, TBI, and TLE (Liu et al., 2023; Ma et al., 2022; Norton et al., 2021; Zhang et al., 2022). *Serpina3n* was found to be upregulated following the kainate (KA)-induced seizure model and was shown to mediate immune responses in astrocytes (Liu et al., 2023). While its overexpression increased expression of multiple cytokines, silencing of Serpina3n reduced cytokine expression and NFκB signaling (Liu et al., 2023). Dysregulation of these genes may suggest that astrocyte reactivity and immune response are altered in ephrin-B1 KO mice, which is supported by our finding that GFAP immunoreactivity was increased in the corpus callosum of KO mice.

Additionally, we observed that genes associated with calcium signaling in astrocytes were significantly upregulated, such as *Gpm6a* and *Cpne2* (Figure 1E). In neurons, Gpm6a has been implicated in NGF-mediated Ca^2+^ influx (Mukobata et al., 2002). In addition, *Gpm6a* was shown to bind to many synaptic proteins, and of interest to this study, was also found to bind to Plp, one of the major myelin proteins (Aparicio et al., 2020). *Gpm6a* expression has been detected in subsets of astrocytes in a mouse model of TBI, however the function of Gpm6a remains to be explored (Choi et al., 2013). Interestingly, the most significantly regulated gene we detected via Nanostring analysis was the Slc8a1 gene (Figure 1E), encoding NCX sodium-calcium exchanger. Expression of this transporter in astrocytes is hypothesized to convert sodium transients induced by excitatory neuronal activity into astrocyte calcium signals through reverse transport of calcium through the exchanger (Rose et al., 2020). The increased expression of *Slc8a1* in KO animals, therefore, could suggest that astrocytes are more responsive to excitatory neuronal activity and are able to initiate calcium signaling in response to more modest neuronal activity. Astrocyte calcium signaling is also known to induce the release of gliotransmitters such as D-serine, adenosine, ATP, and GABA, therefore alterations in astrocyte calcium signaling could have profound effects on neurotransmission and neuronal excitability (Bazargani & Attwell, 2016; Goenaga et al., 2023).

Most importantly, we found that genes associated with oligodendrocyte development, myelination, and lipid metabolism were all significantly decreased in KO animals (Figure 2B-D). For example, mRNA levels of *Dlx1*, a negative regulator of oligodendroglial versus neuronal specification during development (Petryniak et al., 2007) were reduced in KO mice (Figure 2B). Furthermore, mRNA levels of *Sox10*, one of the major drivers of oligodendrocyte differentiation and a transcription factor required for oligodendrocytes to acquire a myelinating phenotype was also significantly reduced in KO animals (Figure 2B) (Kuhn et al., 2019; Pozniak et al., 2010; Stolt et al., 2002), suggesting that oligodendrocyte development was altered in KO mice. Only mature oligodendrocytes acquire the ability to myelinate axons and begin expressing myelin proteins such as Plp, MBP, MAG, and MOG, therefore the reduced mRNA levels of all of these genes also suggest impaired oligodendrocyte maturation in KO mice as well as reduced myelination (Kuhn et al., 2019). We also detected decreases in several genes associated with lipid metabolism (Figure 2D), including cholesterol, ceramide, and sphingolipid metabolism, all of which are major components of the myelin sheath (Chrast et al., 2011; Coetzee et al., 1998; Eckhardt, 2023; Ho et al., 2022; Maldonado et al., 2008; Marcus et al., 2000; Poitelon et al., 2020; Stadelmann et al., 2019). Altogether, Nanostring mRNA analysis revealed a marked downregulation of many oligodendrocyte, myelin, and lipid metabolism genes in KO animals, which suggests a supportive role for astrocytic ephrin-B1 in oligodendrocyte development and myelination during development.

To address whether the changes in oligodendrocyte and myelin related genes resulted in changes in oligodendrocyte development or myelination at the protein level, we used immunofluorescence staining to detect the number of oligodendrocytes and the MBP levels in the corpus callosum, one of the largest white matter tracts in the brain, and the hippocampus (Figure 3). In support of our gene expression data, we found that both the number of oligodendrocytes and the MBP levels were reduced in the CC of KO mice (Figure 3A-F, M-O), corroborating the mRNA changes detected by Nanostring analysis and suggesting that oligodendrocyte development and myelination were impaired in KO mice. The changes were less robust in the hippocampus, possibly due to the fact that there were fewer oligodendrocytes in the hippocampus than the CC. The data supports that astrocytic ephrin-B1 regulates oligodendrocyte development and myelination during the P14-P28 developmental period. To test if the observed deficits in oligodendrocyte numbers and myelination resulted in functional impairments in vivo, we tested whether KO mice exhibited a clasping phenotype and impaired rotarod performance, indicative of reduced motor strength due to impaired myelination. Indeed, developmental deletion of astrocytic ephrin-B1 resulted in an increase in clasping behavior (Figure 4A-D) and impaired rotarod performance (Figure 4E). Previous studies showed that demyelination induced by EAE and Sod2 deletion in motor neurons resulted in clasping behavior, suggesting that the observed clasping phenotype can be attributed to poor myelination (Bhaskaran et al., 2023; Cahill et al., 2019).

Astrocytes and oligodendrocytes are functionally coupled through pairs of connexin (Cx) gap junction proteins, allowing for electrical coupling, spatial buffering, and transfer of metabolites between the cells (Stadelmann et al., 2019). Astrocytes express Cx30 and Cx43, while oligodendrocytes express Cx47 and Cx32 and astrocyte-oligodendrocyte gap junctions are composed of Cx47/Cx43 or Cx32/Cx30 pairs, where Cx32/30 channels are localized to the outer layer of myelin sheaths, while Cx47/Cx43 channels are more often localized to oligodendrocyte somata (Altevogt & Paul, 2004; Kamasawa et al., 2005; Kleopa et al., 2004; Stadelmann et al., 2019). Interestingly, Nanostring analysis revealed that mRNA levels of Cx-32 were reduced in KO animals (Figure 2E). Further qPCR analysis revealed that mRNA levels of the partner for Cx32 expressed on astrocytes, Cx30, was also significantly reduced in KO animals (Figure 2E). However, mRNA levels of Cx43 were not affected by developmental deletion of astrocytic ephrin-B1 (Figure 2E). These findings are intriguing because they suggest that impaired astrocyte-oligodendrocyte communication likely occurs at the myelin sheath but not necessarily at the oligodendrocyte soma in our model. Other groups showed that double deletion of Cx47/Cx30 impaired myelin formation and resulted in thinner myelin sheaths (Menichella et al., 2003; Tress et al., 2012), therefore downregulation of Cx32/30 in KO mice could explain the observed impairments in myelination. It was also shown that lipid synthesis in astrocytes is required for proper myelination (Camargo et al., 2017) and that cholesterol transfer from astrocytes to oligodendrocytes regulates oligodendrocyte survival and remyelination (Molina-Gonzalez et al., 2023). Since gap junctions allow for metabolite transfer between astrocytes and oligodendrocytes, it is conceivable that the observed impairments in myelination and lipid metabolism in KO animals can be at least partially explained by the downregulation of Cx32 and Cx30. Future studies should address whether impaired coupling between astrocytes and oligodendrocytes is responsible for the observed deficits and if restoring levels of Cx32 in oligodendrocytes and/or Cx30 in astrocytes would improve astrocyte-oligodendrocyte communication and rescue the deficits in oligodendrocyte development, myelination and motor strength in KO mice. Alternatively, an indirect mechanism may explain the changes in myelination. Neuronal activity is intimately linked with both myelination and oligodendrocyte proliferation (Barres & Raff, 1993; Demerens et al., 1996; Gautier et al., 2015; Gibson et al., 2014; Mitew et al., 2018; Ortiz et al., 2019). Indeed, previous work in our lab showed that astrocytic ephrin-B1 negatively regulates excitatory neurotransmission and synaptogenesis while positively regulating inhibitory neurotransmission (Nguyen et al., 2020). Therefore, the impaired myelination in KO mice may also be due to the previously observed increase in neuronal activity and E/I balance. In addition, increased expression of the cell surface receptor *Amigo-2*, which is associated with A1-polarized reactive astrocytes (Kim et al., 2024), can also affect oligodendrocyte development and myelination. Indeed, we observed increased GFAP immunoreactivity in the corpus callosum, but not hippocampus following developmental deletion of ephrin-B1 from astrocytes, which may potentially be responsible for the observed changes in the genes associated with oligodendrocyte development, myelination, and lipid metabolism. GFAP positive astrocytes are also known positively regulate oligodendrocytes development and myelination as their ablation was previously shown to reduce the number of mature oligodendrocytes and myelin formation in the developing spinal cord (Tognatta et al., 2020). Future work will investigate whether the changes in myelination are due to direct interactions involving connexin proteins or EphB signaling in oligodendrocytes or are due to indirect mechanisms involving changes in astrocytes phenotype or neuronal activity.

Our study shows for the first time that astrocytes contribute to oligodendrocyte development and myelination through ephrin-B1 signaling. Deletion of astrocytic ephrin-B1 reduced gene expression levels of genes associated with oligodendrocyte development, myelin, and lipid metabolism. In addition, this led to reduced numbers of oligodendrocytes and reduced myelination, resulting in a clasping phenotype and impaired performance in the rotarod test. Future studies can address whether impaired ephrin-B1 expression in astrocytes is associated with demyelinating diseases such as MS and explore whether astrocytic ephrin-B1 may be useful as a therapeutic target to promote remyelination and oligodendrocyte survival in demyelinating diseases.

## Supplementary Material

Supplemental Material

Supplemental Material

Supplemental Material

Supplemental Material
