# Supplementary material for: Astrocytic Ephrin-B1 Regulates Oligodendrocyte Development and Myelination
Source: ASN Neuro. 2024 Oct 22;16(1):2401753. doi: 10.1080/17590914.2024.2401753 (PMC11792131; doi:10.1080/17590914.2024.2401753)
Supplement: Supplemental Material [file TASN_A_2401753_SM5828.docx]

**Extended Data for Fig. 1C:**

|  | **Ephrin-B1** |
| --- | --- |
| **CON** | 54.08 ± 4.73 |
| **KO** | 21.21 ± 6.18 |
| **Statistics** | t_(18)_ = 4.280  p = 0.0005 |
