# Supplementary material for: Astrocytic Ephrin-B1 Regulates Oligodendrocyte Development and Myelination
Source: ASN Neuro. 2024 Oct 22;16(1):2401753. doi: 10.1080/17590914.2024.2401753 (PMC11792131; doi:10.1080/17590914.2024.2401753)
Supplement: Supplemental Material [file TASN_A_2401753_SM5515.docx]

**Extended Data for Fig. 2C:**

| Gene | Forward Primer | Reverse Primer |
| --- | --- | --- |
| Connexin 43 (Gja1) | 5′-GTGCCGGCTTCACTTTCATT-3′ | 5′-CGTGGAGTAGGCTTGGACCTT-3′ |
| Connexin 30 (Gjb6) | 5′-GCAGAGGGATTTTGCAGTGACT-3′ | 5′- TGTTCACGCCACCGATGA-3′ |

|  | **Cx30** | **Cx43** |
| --- | --- | --- |
| **CON** | 1.005 ± 0.03721 | 1.017 ± 0.02291 |
| **KO** | 0.8372 ± 0.04223 | 1.044 ± 0.01543 |
| **Statistics** | t_(4)_ = 2.98  p = 0.0407 | t_(4)_ = 1.005  p = 0.3719 |
