# Supplementary material for: Astrocytic Ephrin-B1 Regulates Oligodendrocyte Development and Myelination
Source: ASN Neuro. 2024 Oct 22;16(1):2401753. doi: 10.1080/17590914.2024.2401753 (PMC11792131; doi:10.1080/17590914.2024.2401753)
Supplement: Supplemental Material [file TASN_A_2401753_SM5514.docx]

**Extended Data for Fig. 3:**

|  | **MBP levels in CC** | **Olig2 Cell Count in CC** | **MBP levels in Hippocampus** | **Olig2 Cell Count in Hippocampus** |
| --- | --- | --- | --- | --- |
| **CON** | 19.86 ± 3.063 | 160.1 ± 13.20 | 1.524 ± 0.3544 | 56.53 ± 4.113 |
| **KO** | 8.764 ± 1.635 | 104.1 ± 7.777 | 1.785 ± 0.2323 | 46.44 ± 3.980 |
| **Statistics** | t_(6)_ = 3.197  p = 0.0187 | t_(14)_ = 3.656  p = 0.0026 | t_(6)_ = 0.6161  p = 0.5605 | t_(29)_ = 1.764,  p = 0.0883 |
