# Supplementary material for: Astrocytic Ephrin-B1 Regulates Oligodendrocyte Development and Myelination
Source: ASN Neuro. 2024 Oct 22;16(1):2401753. doi: 10.1080/17590914.2024.2401753 (PMC11792131; doi:10.1080/17590914.2024.2401753)
Supplement: Supplemental Material [file TASN_A_2401753_SM5513.docx]

**Extended Data for Fig. 4A-C**

|  | **Clasping at 10 s** | **Clasping at 30 s** | **Clasping at 60 s** |
| --- | --- | --- | --- |
| **CON** | 0 ± 0 | 0 ± 0 | 0.2000 ± 0.1333 |
| **KO** | 0.8095 ± 0.1905 | 0.9048 ± 0.1677 | 1.048 ± 0.1756 |
| **Statistics** | MWU = 50  p = 0.0066 | MWU = 35  p = 0.0008 | MWU = 44  p = 0.0065 |

**Extended Data Fig. 4D**

| **Log Rank Test** |  |
| --- | --- |
| χ^2^ | 7.963 |
| DoF | 1 |
| P Value | 0.0048 |
| Hazard Ratio CON/KO | 0.1994 |
| 95% CI of ratio CON/KO | 0.07557 to 0.5259 |
| Hazard Ratio KO/CON | 5.016 |
| 95% CI of ratio KO/CON | 1.901 to 13.23 |
